# LRG1 downregulation in allergic airway disorders and its expression in peripheral blood and tissue cells

**DOI:** 10.1186/s12967-016-0929-2

**Published:** 2016-07-04

**Authors:** Lijing Hao, Hua Xie, Bin Zhang, Dong Chen, Shufen Wang, Huiyun Zhang, Shaoheng He

**Affiliations:** Allergy and Clinical Immunology Research Centre, The First Affiliated Hospital of Jinzhou Medical University, No. 2, Sect. 5, Renmin Street, Guta District, Jinzhou, 121001 Liaoning People’s Republic of China; Department of Dentistry, The First Affiliated Hospital of Jinzhou Medical University, Jinzhou, 121001 Liaoning People’s Republic of China; Allergy Clinic, The General Hospital of Shenyang Military Region, Shenyang, 110016 Liaoning People’s Republic of China

**Keywords:** LRG1, TGFBR2, Allergic airway disorder, Mast cells, Leukocytes

## Abstract

**Background:**

Increased leucine-rich α2-glycoprotein-1 (LRG1) has been observed in plasma of individuals with various diseases. However, the role of LRG1 in allergic airway disease has not been investigated.

**Objective:**

To explore the involvement of LRG1 in allergy and its cell origins.

**Methods:**

The expression levels of LRG1 and its receptor transforming growth factor-beta receptor II (TGFBR2) in patients with allergic rhinitis (AR) and asthma (AS) were examined by flow cytometry, and enzyme-linked immunosorbent assay (ELISA).

**Results:**

LRG1 and soluble TGFBR2 expression in plasma of patients with AR and AS were markedly lower than that of healthy control (HC) subjects. Large proportions of CD123 + HLA-DR−, CD16+, CD4+, CD8+, CD14+, and CD19+ cells expressed LRG1, although the percentages of LRG1+ cells in these cell populations were lower in AR and AS patients. Up to 89.8 and 15.5 % of dispersed mast cells expressed LRG1 and TGFBR2. Moreover, allergen extract exposure significantly reduced LRG1 and TGFBR2 expression in the plasma and leukocytes of patients with AR and AS.

**Conclusions:**

Reduced LRG1 and TGFBR2 levels in patients with allergic airway disorders are likely caused by inhibitory actions of allergens in LRG1 producing cells. Thus, LRG1 may be a key regulatory factor of allergic responses.

## Background

LRG1 (*HGNC: 29480*) is a 50-kDa glycoprotein containing 23 % carbohydrate by weight [1]. It consists of 312 amino acids, of which 66 are leucines. The predicted secondary structure suggests that LRG1 may be a membrane-associated or membrane-derived protein [2]. LRG1 belongs to the leucine-rich repeat protein family [3], members of which are commonly involved in signal transduction, cell adhesion, development, DNA repair, recombination, transcription, and RNA processing [4].

LRG1 is expressed during haematopoiesis, especially during differentiation of the neutrophilic granulocyte lineage [5]. It also promotes neovascularization through binding to the accessory receptor endoglin, and activates transforming growth factor beta (TGF-β) signalling in endothelial cells via the TGFBR2 (*HGNC: 11773*) -ALK-1/Smad1/5/8 pathway [6]. Increased LRG1 levels have been observed in serum or plasma of patients with various types of cancers [7] including lung cancer [8]. It is suggested that LRG1 may be a useful marker of pediatric appendicitis as it was elevated in urine [9] and plasma [10] of children with acute appendicitis and enriched in diseased appendices. Because serum LRG concentrations correlate well with disease activity in ulcerative colitis [11] and rheumatoid arthritis (RA) [12], this novel serum biomarker could be a promising surrogate for C-reactive protein when monitoring disease progression in ulcerative colitis and RA. However, little is known regarding the relationship between this unique glycoprotein and allergic disorders. Since allergy is also an inflammatory and immunological disease, and LRG may play a role in it, we investigated the expression of LRG1 in allergic disorders and its potential cell origins in the present study.

LRG1 is co-expressed with TGFBR2 and contains a putative membrane-binding region, thus suggesting TGFBR2 as a potential cell-surface receptor for LRG1 [13]. Elevated levels of TGFBR2 and LRG1 are observed in the cerebrospinal fluid of patients with idiopathic normal pressure hydrocephalus (INPH) [14], suggesting measuring TGFBR2 and LRG1expression levels may be useful for diagnosing INPH. However, the expression levels of TGFBR2 in the plasma and blood cells under allergic conditions remain uninvestigated. The aim of the study is to investigate the expression levels of LRG1 and its receptor TGFBR2 in the plasma and blood cells under allergic airway conditions.

## Methods

### Reagents

Type-I collagenase, and type-I hyaluronidase were purchased from Sigma-Aldrich (St Louis, MO, USA). FACS lysing solution and Cytofix/Cytoperm solution were obtained from BD Pharmingen (San Jose, CA, USA). A rabbit anti-human LRG1 antibody, a FITC-conjugated mouse anti-rabbit IgG antibody, and a PE-conjugated mouse anti-rabbit IgG antibody were purchased from LifeSpan BioSciences (Seattle, WA, USA). PerCP-conjugated mouse anti-human CD4, PE/Cy7-conjugated mouse anti-human CD8, APC/CY7-conjugated mouse anti-human CD14, APC-conjugated mouse anti-human CD19, APC/Cy7-conjugated mouse anti-human CD16, PE/Cy7-conjugated mouse anti-human CD123, PerCP-conjugated mouse anti-human HLA-DR, PE/Cy7-conjugated mouse anti-human CD34, PerCP-conjugated mouse anti-human FcεR1, PE-conjugated mouse anti-human CD117, FITC-conjugated mouse anti-human CD90, APC-conjugated mouse anti-human TGFBR2, and PE-conjugated mouse anti-human TGFBR2 antibodies were purchased from BioLegend (San Diego, CA, USA). Allergens for skin prick tests were supplied by ALK-Abelló, Inc. (Denmark). Human LRG1, TGFBR2, and tryptase ELISA kits were purchased from R&D Systems (Minneapolis, MN). Most general-purpose chemicals such as salts and buffer components were of analytical grade.

### Patients and samples

A total of 16 AR, 15 AS, 5 combined AR and AS (AR + AS), 8 RA and 15 HC subjects were recruited for the study. AS was diagnosed according to the criteria of the Global Initiative for Asthma [15], and AR and RA diagnosis were based on the allergic rhinitis and its impact on asthma [16] and 2010 RA classification criteria [17], respectively. All patients were asked to stop taking anti-allergy medication for at least 2 weeks prior to participating in the study. The recruited patients did not have any airway infection for >1 month. Informed consent was provided from each volunteer according to the declaration of Helsinki, and this study was approved by the ethical committees of the First Affiliated Hospital of Jinzhou Medical University. The general characteristics of the patients and control subjects are summarized in Table 1. Peripheral venous blood sample was collected into K2EDTA anticoagulant containing tubes from each patient and HC subject, and were immediately processed to collect cells and plasma for analysis. Human tonsillar specimen tissues were removed by tonsillectomy, after which dispersing mast cells and fibroblasts were collected. The protocol for the ethical use of human tissue in research complied with the Declaration of Helsinki (2000) and was approved by the Committees of the First Affiliated Hospital of Jinzhou Medical University.

**Table 1:** General characteristics of the patients with rhinitis (AR), asthma (AS), combined rhinitis with asthma (AR + AS), rheumatoid arthritis (RA) or healthy control subjects (HC)

	HC	AR	AS	AR + AS	RA
	(n = 15)	(n = 16)	(n = 15)	(n = 5)	(n = 8)
Age (year)	35 (15–65)	43(17–74)	47 (18–78)	43 (16–60)	48 (20–68)
Female/male	8/7	9/7	7/8	3/2	7/1
Median age at onset (year)	na	36 (7–56)	42 (10–59)	43 (6–57)	40 (18–63)
Median disease duration (year)	na	3.5 (0.5–21)	3 (0.5–38)	5 (1–40)	4 (0.5–42)
Blood taken time after the first symptom of the latest attack (h)	na	12 (5–18)	9 (4–17)	8 (3–16)	12 (6–16)
Number of positive skin prick for house dust mite	0	12	10	3	0
Number of positive skin prick for artemisia	0	9	3	1	0
Number of positive skin prick for platanus pollen	0	2	5	1	0
Number of positive skin prick for cat fur	0	4	4	3	0
Number of positive skin prick for dog fur	0	5	3	0	0

Median (range) data are shown for the number of subjects indicated. All patients stop using long-acting corticosteroids for at least 2 weeks and any other anti-allergic drugs for 1 week before skin prick being taken

*na* not applicable

### Cell line and culture

A human mast cell line, HMC-1, was a gift from Dr. Joseph H. Butterfield (Mayo Clinic, MN, USA). Cells were cultured in RPMI 1640 medium supplemented with 10 % fetal bovine serum and 1 % penicillin and streptomycin in 75-cm^2^ tissue culture flasks (Falcon) at 37 °C in a 5 % (v/v) CO_2_, water-saturated atmosphere.

### Flow cytometric analysis of LRG1 and TGFBR2 expression in white blood cells from patients exposed to allergens

To detect LRG1 and TGFBR2 expression on basophilic granulocytes (CD123 + HLA-DR− cells) and neutrophils (CD16+ cells), we added 4 antibodies (APC/CY7-conjugated mouse anti-human CD16, PE/CY7-conjugated mouse anti-human CD123, PerCP-conjugated mouse anti-human HLA-DR, and PE-conjugated mouse anti-human TGFBR2) to 100 μl whole blood, and the cells were labelled for 15 min, according to the manufacturer’s instructions. Following the ligation of red blood cells, white blood cells were fixed and permeabilized. The rabbit anti-human LRG1 antibody were added to 100 μl cell suspensions for 30 min at 4 °C, followed by staining with a FITC-conjugated mouse anti-rabbit IgG antibody. To detect LRG1 and TGFBR2 expression on monocytes (CD14+ cells), helper T cells (CD4+ cells), cytotoxic T cells (CD8+ cells), and B cells (CD19+ cells), we stained cells with the following antibodies: PerCP-conjugated mouse anti-human CD4, PE/Cy7-conjugated mouse anti-human CD8, APC/Cy7-conjugated mouse anti-human CD14, and APC-conjugated mouse anti-human CD19. After washing, the cells were analysed on a fluorescence-activated cell sorting (FACS)Arial flow cytometer with CellDevia software (BD Biosciences, USA).

### Peripheral blood challenge with allergen extracts

Peripheral blood samples (1.5 ml) from 8 AR, 8 AS, and 8 HC subjects were incubated with *Artemisia sieversiana* wild allergen extract (ASWE) at 0.1 and 1.0 μg/ml, *Platanus* pollen allergen extract (PPE) at 0.1 and 1.0 μg/ml, and house dust mite allergen extract (HDME) at 0.1 and 1.0 μg/ml for 30 min at room temperature. The challenged blood was then processed as above.

### Isolation of tissue cells and flow cytometric analysis of LRG1 and TGFBR2 expression

The procedures for dispersing human tonsillar and skin tissue cells were mainly adopted from a previous study by He et al. [18]. Briefly, tonsillar and skin tissues were digested with collagenase, hyaluronidase, and DNase in DMEM. After centrifugation, the cells were fixed using a Cytofix/Cytoperm™ solution. Cells were then incubated with one of the following labelled monoclonal antibodies: PE/Cy7-conjugated mouse anti-human CD34, PerCP-conjugated mouse anti-human FcεR1, PE-conjugated mouse anti-human CD117, FITC-conjugated mouse anti-human CD90, rabbit anti-human LRG1, FITC-conjugated mouse anti-rabbit IgG, PE-conjugated mouse anti-rabbit IgG, or APC-conjugated mouse anti-human TGFBR2, with staining performed for 30 min at 4 °C in the dark. FITC-conjugated mouse IgG1, PE-conjugated mouse IgG1, and APC-conjugated mouse IgG1 were used as isotype control. Cells were analysed on a FACSArial flow cytometer. Data were analysed with CellQuest software.

### Time course of LRG1 and TGFBR2 expression in HMC-1 cells

The procedure challenging HMC-1 cells was mainly adopted from a method previously described by Zhang et al. for P815 cells [19]. Briefly, cultured HMC-1 cells at a density of 1 × 10^6^ cells/ml were incubated with ASWE (0.1 and 1.0 μg/ml), PPE (0.1 and 1.0 μg/ml), or HDME (0.1 and 1.0 μg/ml) for 1, 6, or 12 days at 37 °C, changing the culture medium and allergen at every 2 days. The plates were centrifuged at 450×*g* for 10 min at 4 °C before the culture supernatants (1 ml per well) were collected and stored. Cell pellets containing approximately 1 × 10^6^ cells were resuspended for FACS analysis.

### Determination of the expression levels of LRG1, TGFBR2, and cytokines in the plasma of allergic patients

The levels of tryptase, LRG1, and TGFBR2 produced in the plasma of allergic patients were measured using ELISA kits, according to the manufacturer’s instructions.

### Statistical analysis

All statistical analyses were performed with SPSS software for windows (version 17.0, IBM Corporation). Data were presented as median (range) for the number of experiments indicated. Where analysis of variance indicated significant differences between groups (Kruskal–Wallis test) for pre-planned comparisons of interests, the Mann–Whitney U test was applied. For all analyses, P < 0.05 was considered statistically significant.

## Results

### Plasma levels of LRG1, TGFBR2, and tryptase and their correlations

**Fig. 1:**
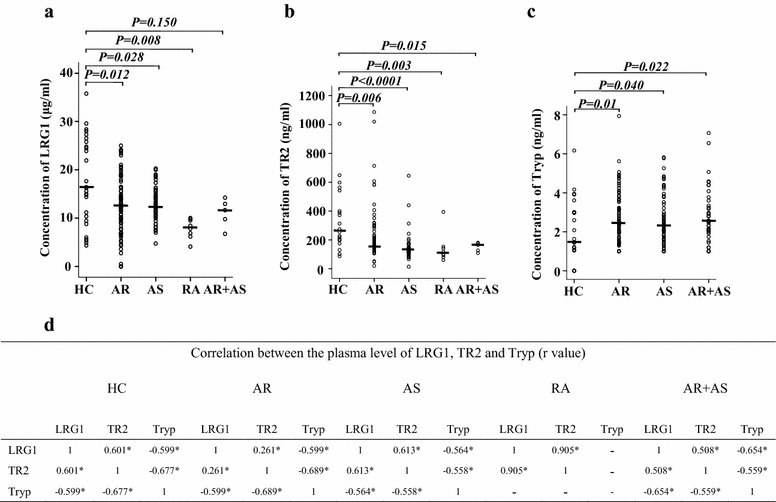
Scatter plots showing the levels of LRG1 (**a**), TGFBR2 (**b**, TR2), and tryptase (**c**, Tryp) in the plasma of patients with allergic rhinitis (AR), asthma (AS), AR + AS, rheumatoid arthritis (RA), and healthy control (HC) subjects. Each symbol represents the value from 1 subject. The median value is indicated with a* horizontal line*. **d** Rank correlation (Spearman’s correlation coefficient) between LRG1, TR2, and Tryp. *P < 0.05. *na* not applicable

The most direct approach for studying the potential roles of LRG1 in allergic disorders is to examine changes in LRG1 expression under allergic conditions. RA patients were used as a control disease population. Using ELISA kits, we observed that LRG1 levels in the plasma of AR, AS, and RA subjects, but not AR + AS subjects, were markedly lower than that of HC subjects (Fig. 1a). Similarly, soluble TGFBR2 levels in the plasma of AR, AS, RA, and AR + AS groups were also markedly lower than that of those of the HC group (Fig. 1b). In contrast, tryptase levels in the plasma of AR, AS, and AR + AS groups were markedly higher than those of the HC group (Fig. 1c). LRG1 levels correlated well with TGFBR2 production in the plasma of AR, AS, AR + AS, and HC subjects. Tryptase expression was negatively correlated LRG1 and TGFBR2 in plasma samples from AR, AS, and AR + AS subjects (Fig. 1d). Positive skin allergen-testing result of the AR and AS patient population indicates that not all patients respond to the same allergen, and appears not to associate with the plasma levels of LRG1 and TGFBR2.

### LRG1 and TGFBR2 expression in peripheral blood leukocytes

**Fig. 2:**
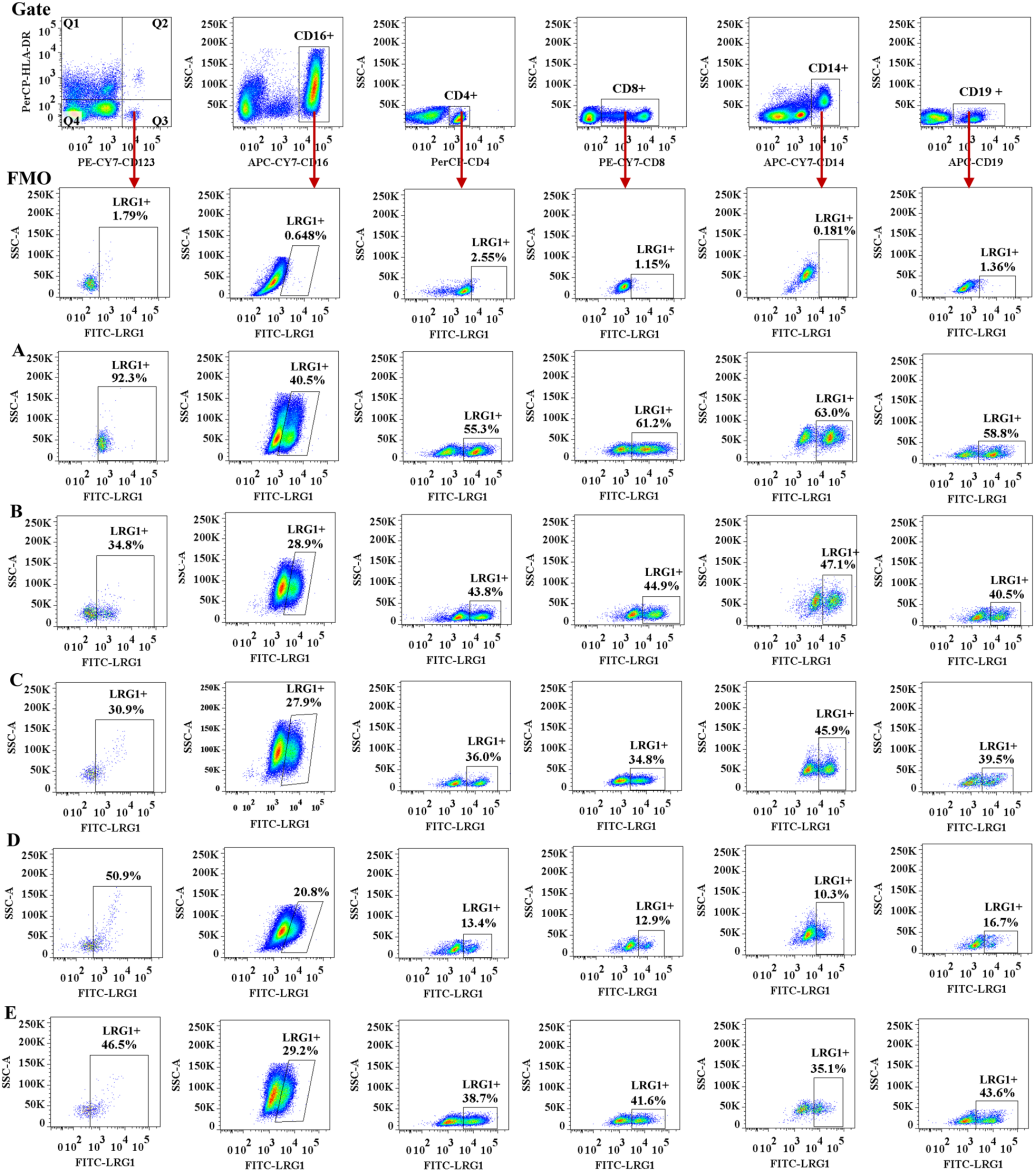
Flow cytometric analysis of LRG1 expression in peripheral blood leukocytes. A representative graph of LRG1+ cells in (A) healthy control (HC) subjects, (B) allergic rhinitis (AR), (C) asthma (AS), (D) rheumatoid arthritis (RA), and (E) AR + AS as indicated. *FMO* fluorescence minus one

**Fig. 3:**
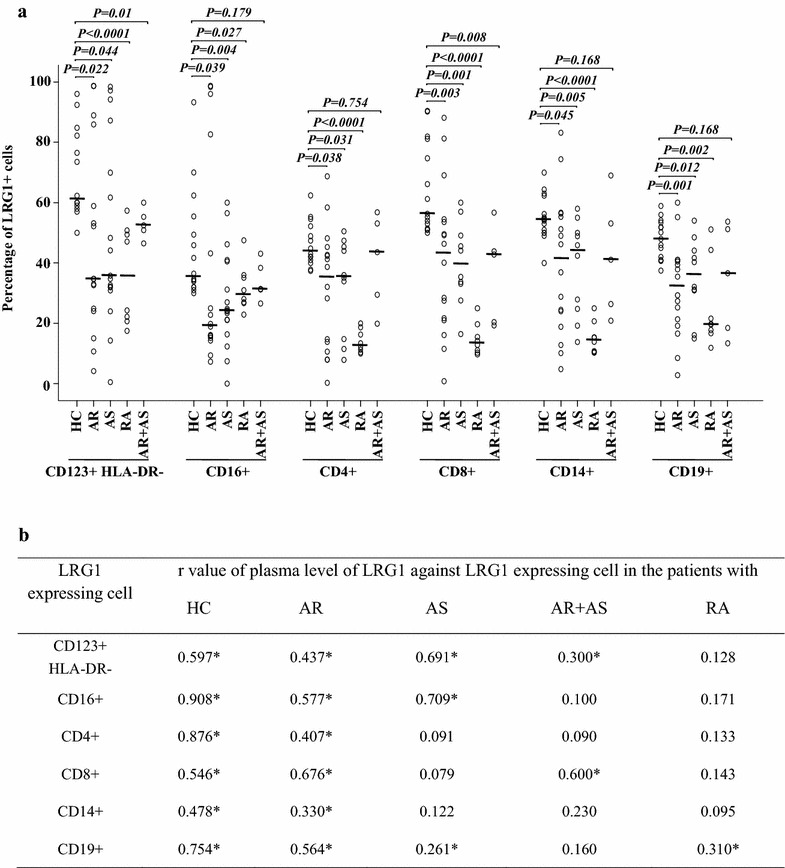
Flow cytometric analysis of LRG1 expression in peripheral blood leukocytes. **a** *Scatter plots* of LRG1 expression. *P* < 0.05 was considered statistically significant. **b** Rank correlation (Spearman’s correlation coefficient) between the plasma level of LRG1 and percentage of LRG1 expressing cells. *P < 0.05

To identify the potential sources of LRG1 and TGFBR2 production, we investigated LRG1 and TGFBR2 expression in peripheral blood leukocytes. The result showed that large proportions of CD123 + HLA-DR−, CD16+, CD4+, CD8+, CD14+, and CD19+ cells expressed LRG1. The percentages of LRG1+ cells in CD123 + HLA-DR−, CD16+, CD4+, CD8+, CD14+, and CD19+ cell populations of AR, AS, and RA patients were lower than those of HC subjects. However, for AR + AS patients, LRG1+ cells were only diminished in CD123 + HLA-DR− and CD8+ cell populations (Fig. 2). No significant difference in the mean fluoresce intensity (MFI) of LRG1 expression was observed between AR, AS, RA or AR + AS, and HC subjects. Decreased LRG1 plasma levels correlated with diminished LRG1 expression in CD123 + HLA-DR−, CD16+, CD4+, CD8+, CD14+, and CD19+ cell populations from AR patients; CD123 + HLA-DR− and CD16 + cells from AS patients; and CD123 + HLA-DR− and CD8+ cells from AR + AS patients (Fig. 3b).

**Fig. 4:**
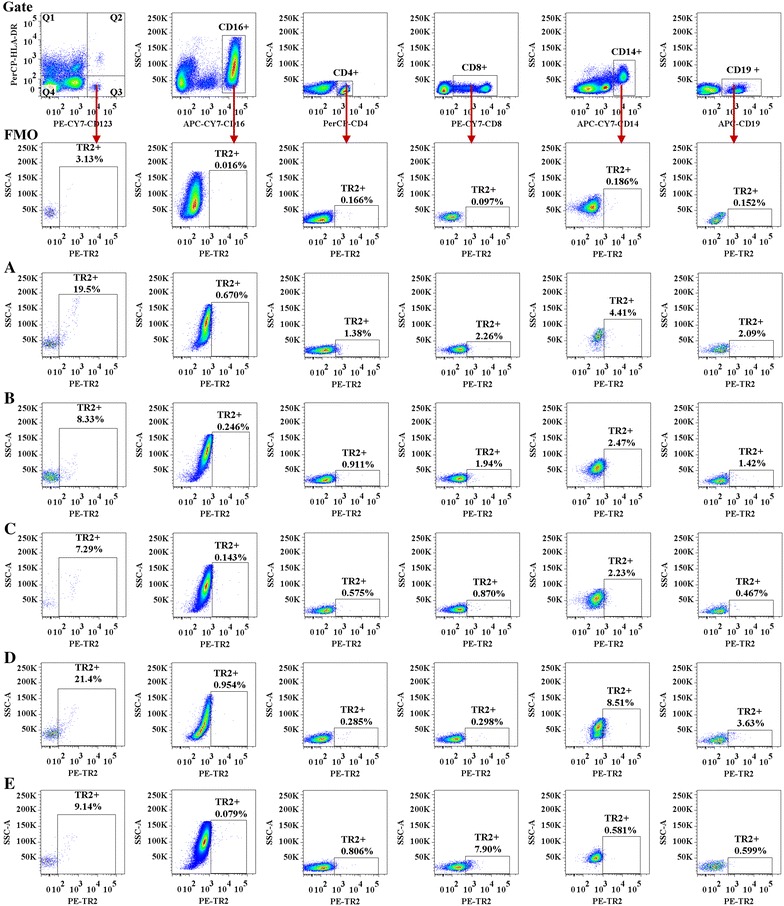
Flow cytometric analysis of TGFBR2 (TR2) expression in peripheral blood leukocytes. A representative graph of TR2+ cells in (A) healthy control (HC) subjects, (B) allergic rhinitis (AR), (C) asthma (AS), (D) rheumatoid arthritis (RA), and (E) AR + AS as indicated. *FMO* fluorescence minus one

**Fig. 5:**
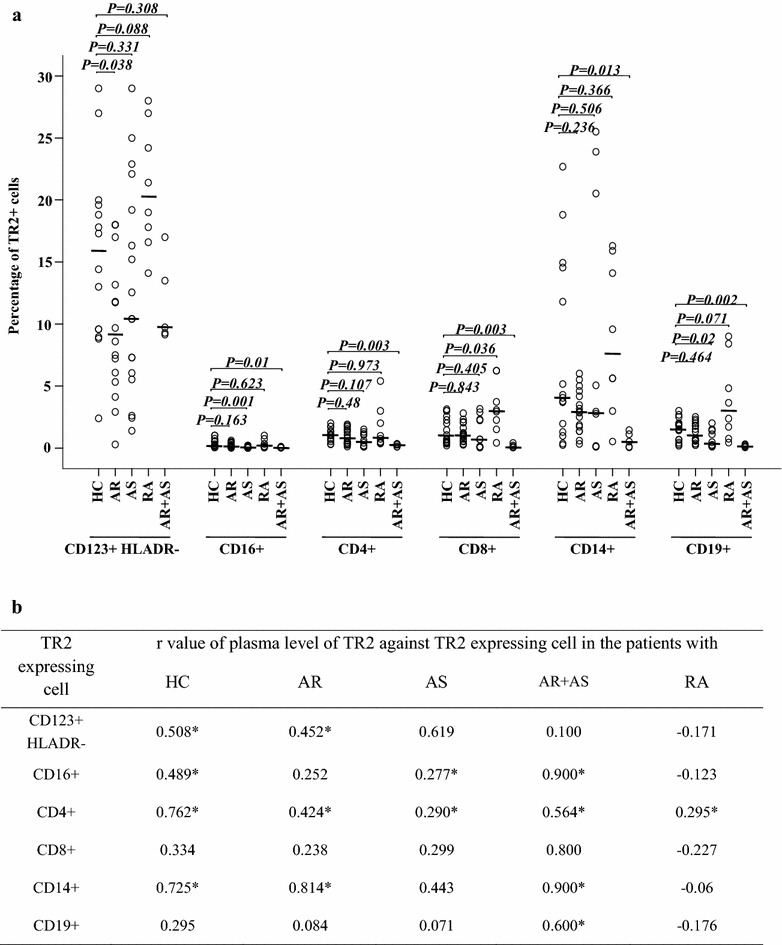
Flow cytometric analysis of TGFBR2 (TR2) expression in peripheral blood leukocytes. **a** *Scatter plots* of TR2 expression. *P* < 0.05 was considered statistically significant. **b** Rank correlation between the plasma level of TR2 and percentage of TR2 expressing cells. *P < 0.05

The percentage of TGFBR2+ cells present in CD123 + HLA-DR− cell populations was lower in patients with AR (median value 9.1 %, P = 0.03) when compared with HC subjects (median value 15.9 %), but not when compared to patients with AS, RA, or AR + AS. It was also observed that the percentages of TGFBR2+ cells in CD16+, CD4+, CD8+, CD14+, and CD19+ cells from the AR + AS group were lower than those from the HC group. Compared with the HC group, the percentage of TGFBR2+ cells in CD19+ cells from the AS group was slightly low (0.36 vs.1.52 %, P = 0.019). In contrast, the percentage of TGFBR2+ cells in the CD14+ cell population from RA subjects was higher than that from HC subjects (Fig. 4). Similar to LRG1, the MFI of TGFBR2 expression was not significantly different between samples from the AR, AS, RA or AR + AS, and HC groups. Decreased plasma levels of TGFBR2 correlated with diminished TGFBR2 expression in the CD123 + HLA-DR−, CD4+ and CD14+ cell populations from the AR group; CD16+ and CD4+ cells from the AS group; CD16+ and CD4+, CD14+, and CD19+ cells from the AR + AS group; and CD4+ cells from the RA group (Fig. 5b).

### Expression of LRG1 and TGFBR2 in dispersed tissue cells

**Fig. 6:**
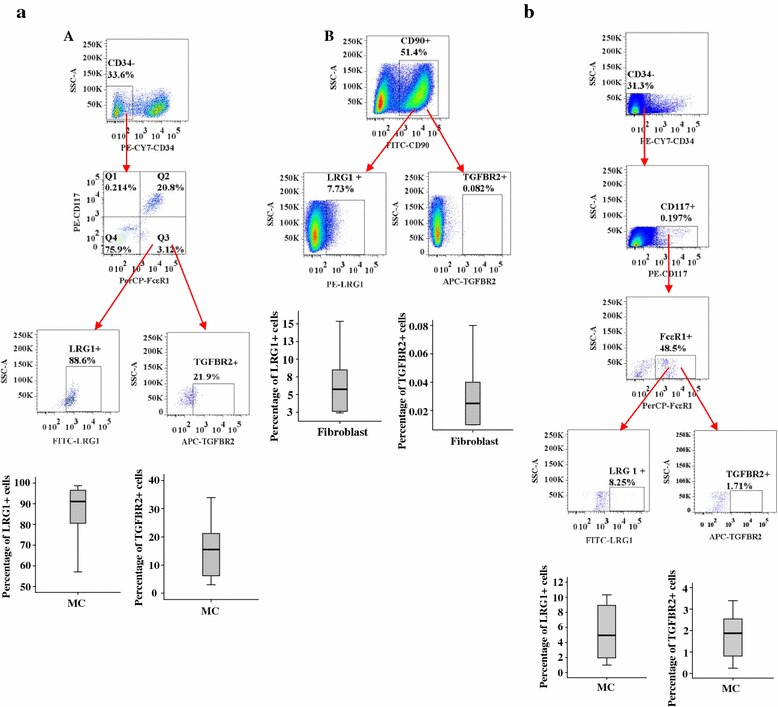
Flow cytometric analysis of LRG1 and TGFBR2 expression in dispersed human mast cells (CD34-CD117 + FcεRI + , MC) and fibroblasts (CD90+). **a** Dispersed skin MC (A) and fibroblasts (B). **b** Dispersed tonsil MC. Data are displayed as a boxplot for 6–7 skin or tonsil tissues, which indicates the median, interquartile range, and the largest and smallest values

To further identify the potential source of LRG1 and TGFBR2 production, we investigated the expression of LRG1 and TGFBR2 in dispersed tissue cells. The result showed that approximately 89.8 % CD34-FcεRI + CD117+ cells (representing mast cells) and 5.7 % CD90+ cells (representing fibroblasts) expressed LRG1, while 15.5 % mast cells and 0.03 % fibroblasts expressed TGFBR2 in skin tissue. In comparison, only 5.0 and 1.9 % mast cells expressed LRG1 and TGFBR2 in tonsil tissue, respectively (Fig. 6).

### Time course of LRG1 expression in HMC-1 cells

**Fig. 7:**
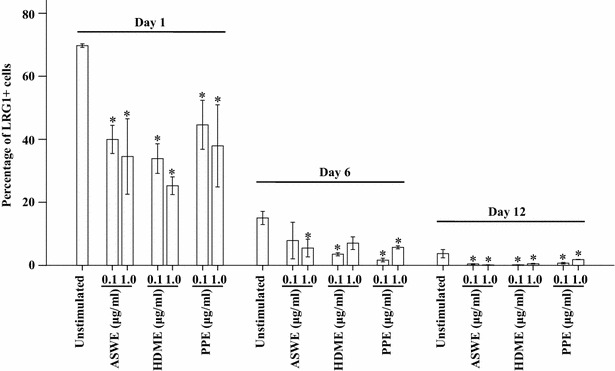
Induction of LRG1 expression in HMC-1 cells by exposure to *Artemisia sieversiana* willd extract (ASWE), *Platanus* pollen extract (PPE), or house dust mite extract (HDME) for 1, 6, or 12 days before being analyzed by flow cytometry. The data are the mean ± SE for 4 separate experiments. *P < 0.05, compared with unstimulated control on the same day

Since reduced plasma level of LRG1 could result from decreased expression of LRG1 in mast cells, we investigated the effects of the ASWE, PPE, and HDME on HMC-1 cells. The result showed that ASWE, PPE or HDME all at 0.1 and 1.0 μg/ml induced dose-dependent downregulation of LRG1 expression in HMC-1 cells on day 1. On day 6 and 12, these allergen extracts also suppressed LRG1 expression in HMC-1 cells. It was also observed that the percentage of LRG1-expressing HMC-1 cells decreased markedly on day 6 and 12, with or without allergen challenge (Fig. 7).

### Decreased levels of LRG1 and TGFBR2 production in plasma following allergen challenge

**Fig. 8:**
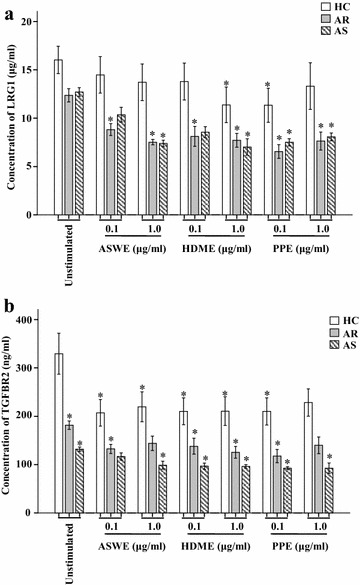
LRG1 (**a**) and TGFBR2 (**b**) production levels in the plasma of allergic rhinitis (AR) and asthma (AS) patient samples following *Artemisia sieversiana* willd extract (ASWE), *Platanus* pollen extract (PPE), or house dust mite extract (HDME) challenge. The values shown are the mean ± SEM for 8 experiments. *P < 0.05 represents statistically different from the corresponding unstimulated group

To further confirm the reduced plasma levels of LRG1 and TGFBR2 in allergic airway disorders, we investigated the effects of the ASWE, PPE, and HDME allergens on LRG1 and TGFBR2 plasma levels. The result showed that ASWE, PPE, and HDME caused up to 40 and 42.2, 48 and 40, and 38.9 and 43.9 % reduction of plasma LRG1 levels in patients with AR and AS, respectively (Fig. 8a). Similarly, ASWE, PPE, and HDME treatment resulted in up to 17.3 and 24.1, 35.9 and 29.7, and 24.1 and 31 % decrease of TGFBR2 plasma levels in patients with AR and AS, respectively (Fig. 8b). Compared with the peripheral blood from HC subjects, challenging the peripheral blood from AR and AS subjects by ASWE, PPE, and HDME reduced the release of LRG1 by up to 54.4 and 55.2, 60.5 and 53.5, and 53.5 and 56.5 %, respectively. Such treatment also reduced the release of TGFBR2 by up to 52.4 and 62.6, 63.1 and 65.4, and 56.3 and 66.0 %, respectively (Fig. 8b).

### Downregulation LRG1 and TGFBR2 expression in peripheral blood leukocytes following allergen challenge

**Fig. 9:**
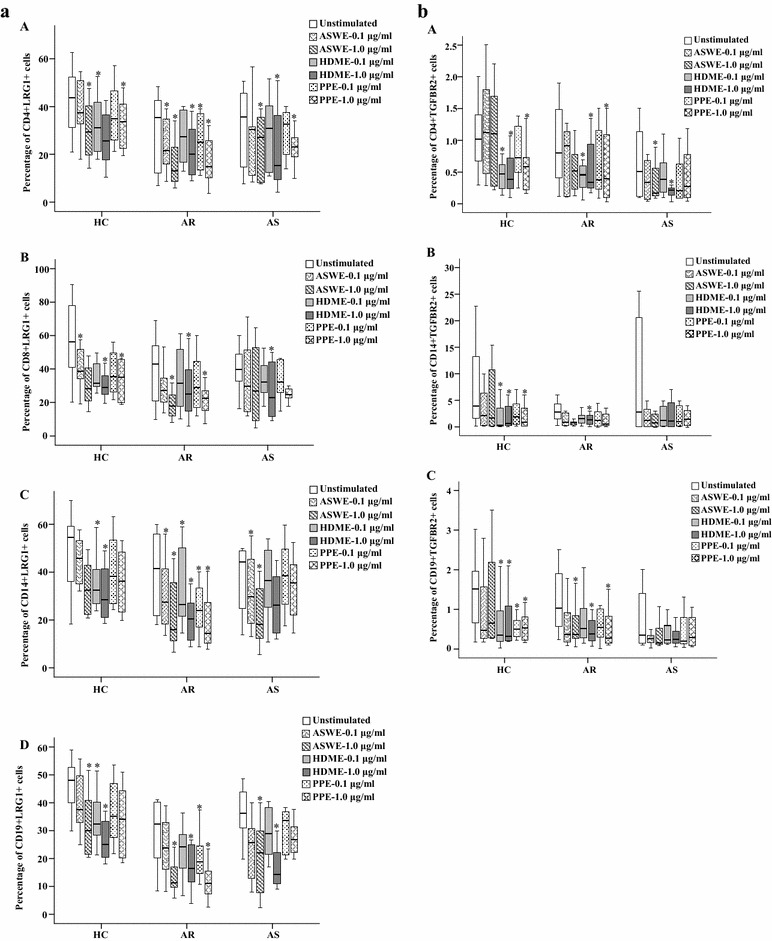
Flow cytometric analysis of LRG1 and TGFBR2 expression in peripheral blood leukocytes of allergic rhinitis (AR, n = 8), asthma (AS, n = 8), and healthy control (HC, n = 8) subjects. **a** LRG1 expression in (A) CD4+, (B) CD8+, (C) CD14+, and (D) CD19+ cells following *Artemisia sieversiana* willd extract (ASWE), *Platanus* pollen extract (PPE) or house dust mite extract (HDME) challenge. **b** TGFBR2 expression in (A) CD4+, (B) CD14+, and (C) CD19+ cells. Data are displayed as a boxplot. *P < 0.05, compared with responses observed in the corresponding unstimulated control

Since allergen-induced reduction of LRG1 and TGFBR2 plasma levels likely resulted from decreased LRG1 and TGFBR2 production in peripheral blood leukocytes, we investigated the effects of the ASWE, PPE, and HDME allergens on LRG1 and TGFBR2 expression in peripheral blood leukocytes. The result showed that ASWE, PPE, and HDME exposure decreased LRG1 expression by up to 62.4, 57.9, and 43; 23.8, 35.2, and 57; and 33.5, 23.3, and 41.7 % in CD4+ cells from AR, AS, and HC subjects, respectively (Fig. 9a). Similarly, ASWE, PPE, and HDME treatment decreased LRG1 expression by up to 57.7, 47.2, and 41.3; 31.7, 37.4, and 41.8; and 49.6, 37.3, and 48.5 % in CD8 + cells from AR, AS, and HC subjects, respectively (Fig. 9a). ASWE, PPE, and HDME decreased LRG1 expression by up to 61.4, 66.1, and 51; 58.7, 19.6, and 40.7; and 40.3, 33.2, and 47.8 % in CD14+ cells from AR, AS, and HC subjects, respectively (Fig. 9a). ASWE, PPE, and HDME treatment also decreased LRG1 expression by up to 64.4, 65, and 48.9; 38.8, 25.7, and 60.2; and 37, 28.7, and 47.7 % in CD19+ cells from AR, AS, and HC subjects, respectively (Fig. 9a). ASWE, PPE, and HDME treatment failed to alter LRG1 expression in CD123 + HLA-DR− and CD16+ cells (data not shown).

Although only up to 1.1, 4.0, and 1.5 % of CD4+, CD14+, and CD19+ cells from the HC group expressed TGFBR2, respectively, HDME and PPE further reduced TGFBR2 expression to 0.4 and 0.6 % in CD4+ cells (Fig. 9b), 0.3 and 0.9 % in CD14+ cells (Fig. 9b), and 0.3 and 0.5 % in CD19+ cells (Fig. 9b), respectively, from the HC group. ASWE, PPE, and HDME treatment also diminished TGFBR2 expression by up to 35.8, 53.1, and 56.8 % in CD4+ cells; up to 73.4, 51.7, and 78.3 % in CD14+ cells; and up to 63.5, 73.1, and 63.5 % in CD19+ cells from the AR group, respectively. ASWE, PPE, and HDME exhibited little effect on TGFBR2 expression in CD123 + HLA-DR−, CD8+, and CD16+ cells (data not shown).

## Discussion

The current study demonstrated for the first time that plasma production levels of LRG1 and its soluble receptor TGFBR2 in AR, AS, and AR + AS subjects are reduced and that LRG1 and TGFBR2 expression is downregulated in various subsets of blood leukocytes and mast cells. Since serum LRG concentrations are increased in autoimmune diseases such as ulcerative colitis [11], RA [12], and various types of cancers, our current findings may provide a novel diagnostic measure for differentiating autoimmune disease or cancer from allergic responses. Because allergens diminish plasma LRG1 and TGFBR2 levels in AR and AS subjects and suppress LRG1 expression in various subsets of blood leukocytes and mast cells, LRG1 likely mediates the pathogenesis of allergic airway disorders.

Observing reduced plasma LRG1 levels in AR, AS and AR + AS subjects was unexpected, as LRG1 downregulation has not been reported previously under pathological conditions. The overwhelming majority of clinical reports have shown that serum or plasma LRG1 levels are elevated during the development of various diseases. For example, LRG1 was upregulated in the serum or plasma of patients with hepatocellular carcinoma [20], pancreatic cancer [21], ovarian cancer [7], lung cancer [8], and colorectal cancer [22]. Increased LRG1 expression was also found in patients undergoing neurodegenerative diseases [23], acute appendicitis [9], hydrocephalus [24], heart failure [25], autoimmune diseases [12], and ageing [26]. LRG1 has also been suggested as promising biomarker in other disease entities, such as Still’s disease [27] and in peptidomics studies [28, 29].

The observation that plasma LRG1 level in patients with RA was significantly decreased was unexpected as previous report [12] demonstrated that LRG level in patients with RA was elevated compared with those in the healthy controls. The main difference between the two studies was the data for HC subjects, which was ~5 μg/ml in the study performed by Serada et al. and 16 μg/ml in the current study. Since different ELISA kits were used by the two study groups, plasma LRG1 was measured in the present study and serum LRG was determined by Serada et al., it is difficult to compare the data from these two studies. Nevertheless, the serum level of LRG for RA in Serada’s study was ~11.5 μg/ml, and the plasma level of LRG1 for RA in our study was 9.2 μg/ml, which seems no big difference between the two.

In contrast to findings with LRG1, some reports have described a decrease of TGFBR2 production under pathological conditions. For instance, decreased TGFBR2 expression was observed in prostate cancer cells [30], carcinoma cells of the urinary bladder [31], and the oropharyngeal mucosa during SIV infection [32]. These data may support our current observation that plasma levels of TGFBR2 were reduced in AR, AS, and AR + AS subjects. It should be noticed that the HC group is significantly younger than the others in the present study, which may be a confounding factor for the interpretation of differences.

LRG1 is expressed during haematopoiesis, especially during differentiation of the neutrophilic granulocyte lineage [5]. We found that not only neutrophils, but also large proportions of basophils, helper T cells, cytotoxic T cells, monocytes, and B cells populations expressed LRG1, which could be the major contributors of plasma LRG1 (μg/ml scale). Thus, reduced plasma LRG1 levels in AR, AS, and AR + AS subjects could result from decreased LRG1 secretion from these subsets of leukocytes. Indeed, we observed diminished percentages of LRG1+ cells in all subsets of leukocytes in patients with AR and AS, in comparison with HC subjects, suggesting that allergens may suppress LRG1 production in these cell types. To our surprise, the plasma level of LRG1 in patients with RA was also lower than that of HC subjects, which conflicts with data from a previous report [12]. Since the plasma LRG1 levels observed in RA patients in the present study is similar to previously reported values (median values: present study, 8.12 vs. previous study, 11.4 μg/ml), the difference between both studies lies in the LRG1 levels observed in HC subjects (median value: present study, 15.9 vs. previous study, 3.0 μg/ml). Since the plasma levels of LRG1 in AR, AS, RA, and AR + AS subjects were all reduced, it is difficult to clearly differentiate AR or AS from RA, based upon plasma LRG1 levels alone.

TGF-β receptors are expressed on almost all types of mammalian cell types examined. The TGF-β signalling pathway involves 2 transmembrane serine/threonine kinases, known as TGF-β receptors I and II [33]. The type-II receptor, a 70 kDa transmembrane protein with a cytoplasm serine/threonine kinase domain, is required for the antiproliferative activity of TGF-β. The abundance of cell-surface TGF-β R-II expression is the limiting factor during initial activation of the signal transduction pathway [34]. Therefore, our findings that plasma TGFBR2 levels were reduced in AR, AS, and AR + AS subjects; that the percentage of TGFBR2+ cells in CD123 + HLA-DR− cells decreased in patients with AR; and that the percentage of TGFBR2 + cells in CD16+, CD4+, CD8+, CD14+, and CD19+ cell populations of AR + AS subjects was lower than that in HC subjects suggested that LRG1 and TGF-β-provoked cell activities may be enhanced in the above cell types.

It was found that ASWE, PPE, and HDME allergen exposure reduced LRG1 and TGFBR2 plasma levels in patients with AR and AS. Because ASWE, PPE, and HDME could also suppress the production of LRG1 and TGFBR2 in peripheral blood helper T cells, NKT cells, monocytes, and B cells from AR and AS subjects, the reduction of plasma LRG1 and TGFBR2 levels may results in part from a decreased release of LRG1 and TGFBR2 from these leukocytes. However, it is difficult to explain the facts that LRG1 concentration was reduced in HC subjects when incubated with 1.0 HDME and 0.1 PPE (and not 1.0 PPE), and that TGFBR2 concentrations were decreased in HC subjects in response to various allergens. Since expression of LRG1 and TGFBR2 are not supposed to be reduced in HC subjects when incubated with allergens, further work is required to address the issue. Lack of association between allergen type in skin allergen-testing and the plasma levels of LRG1 and TGFBR2 in the AR and AS patient population, and 3 different allergens caused similar pattern of reduced LRG1 and TGFBR2 concentrations in plasma, we believe that reduction of plasma levels of LRG1 and TGFBR2 is a common feature of allergy regardless of allergen type.

Little is known of expression of LRG1 in mast cells, the major primary effector cells of allergic responses [35]. Since mast cell degranulation is a key event occurring during allergic responses, the negative correlations between tryptase and LRG1 and TGFBR2 in the plasma of AR, AS, and AR + AS subjects suggested that plasma LRG1 and TGFBR2 may be released from cells other than tryptase-producing cells, or that allergen may have dual activities in mast cells (provoking degranulation and inhibiting LRG1 and TGFBR2 production). Our observations that approximately 89.8 and 15.5 % of mast cells expressed LRG1 and TGFBR2 in skin tissue; that 5.0 and 1.9 % of mast cells expressed TGFBR2 in tonsil tissue; and that the allergens ASWE, PPE, and HDME diminished LRG1 expression in HMC-1 cells support the possibility that allergens may serve such dual roles in mast cells.

## Conclusions

Unlike RA and ulcerative colitis, patients with allergic airway disorders possess a feature of decreased LRG1 concentration in their plasma, which may serve as a maker to differentiate allergic disease from autoimmune disease. The reduced LRG1 and TGFBR2 levels in plasma of allergic airway disorders are most likely caused by the inhibitory actions of allergens on LRG1 and TGFBR2 producing cells. Therefore, LRG1 could be a key regulatory factor of allergy, and its reduced release may contribute to the development of allergy.

## Abbreviations

AR: allergic rhinitis
AS: asthma
ASWE: *Artemisia sieversiana* willd allergen extract
ELISA: enzyme-linked immunosorbent assay
HC: healthy control
HDME: house dust mite allergen extract
HMC-1: human mast cell-1
INPH: idiopathic normal pressure hydrocephalus
LRG1: leucine-rich α2-glycoprotein-1
PPE: *Platanus* pollen allergen extract
RA: rheumatoid arthritis
TR2: TGFBR2 (transforming growth factor-beta receptor II)
